# Beyond a Measure of Liver Function—Bilirubin Acts as a Potential Cardiovascular Protector in Chronic Kidney Disease Patients

**DOI:** 10.3390/ijms20010117

**Published:** 2018-12-29

**Authors:** Ming-Tsun Tsai, Der-Cherng Tarng

**Affiliations:** 1 Division of Nephrology, Department of Medicine, Taipei Veterans General Hospital, Taipei 11217, Taiwan; mingtsun74@gmail.com; 2 Institute of Clinical Medicine, National Yang-Ming University, Taipei 11217, Taiwan; 3 Department and Institute of Physiology, National Yang-Ming University, Taipei 11217, Taiwan

**Keywords:** antioxidant, bilirubin, cardiovascular disease, chronic kidney disease, oxidative stress

## Abstract

Bilirubin is a well-known neurotoxin in newborn infants; however, current evidence has shown that a higher serum bilirubin concentration in physiological ranges is associated with a lower risk for the development and progression of both chronic kidney disease (CKD) and cardiovascular disease (CVD) in adults. The protective mechanisms of bilirubin in CVD, CKD, and associated mortality may be ascribed to its antioxidant and anti-inflammatory properties. Bilirubin further improves insulin sensitivity, reduces low-density lipoprotein cholesterol levels and inhibits platelet activation in at-risk individuals. These effects are expected to maintain normal vascular homeostasis and thus reduce the incidence of CKD and the risks of cardiovascular complications and death. In this review, we highlight the recent advances in the biological actions of bilirubin in the pathogenesis of CVD and CKD progression, and further propose that targeting bilirubin metabolism could be a potential approach to ameliorate morbidity and mortality in CKD patients.

## 1. Introduction

**Figure 1:**
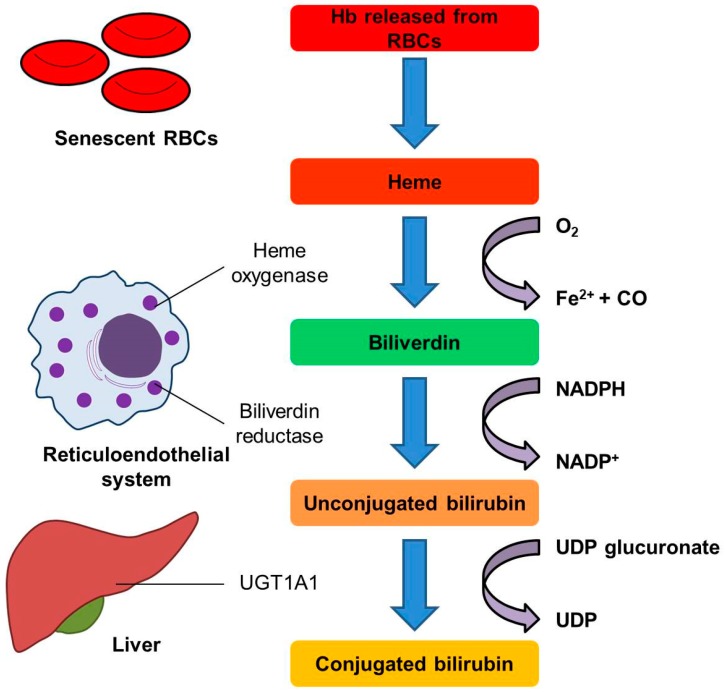
Schematic representation of bilirubin metabolism. Heme released from senescent red blood cells can be readily oxidized by heme oxygenase to biliverdin, which is subsequently reduced to bilirubin by biliverdin reductase. Biliverdin reductase is found in all tissues under physiological conditions, but especially in reticuloendothelial macrophages of the kidney, spleen, liver and brain [5]. Bilirubin binds to albumin and is then transported to the liver, where it is conjugated by UGT1A1 with UDP-glucuronic acid to increase its solubility in water. Abbreviations: CO, carbon monoxide; NADP^+^, nicotinamide adenine dinucleotide phosphate; NADPH, the reduced form of NADP^+^; RBCs, red blood cells; UDP, uridine diphosphate; UGT1A1; uridine diphosphate-glucuronyl transferase 1A1.

Bilirubin is produced by the breakdown of heme, a component mostly derived from the hemoglobin of red blood cells or from other hemoproteins, such as myoglobin, cytochromes, and catalase. Heme oxygenase (HO), a rate-limiting enzyme in heme catabolism, cleaves heme to form biliverdin, which is subsequently converted by biliverdin reductase into bilirubin [1,2]. Bilirubin binds to albumin in the circulation and is transported to the liver, where it is conjugated by uridine diphosphate-glucuronyl transferase 1A1 (UGT1A1) with glucuronic acid and excreted into bile mediated by multi-drug resistance protein-2 (Figure 1) [3,4]. Once entering the bile canaliculi, conjugated bilirubin is stored and mixed with the other constituents of bile in the gall bladder. When eating, the stored bile is propelled into the duodenum to facilitate chemical digestion.

Bilirubin was generally considered as a useless metabolite, with little physiological function, and can be toxic at very high levels. Gamaleldin et al. have shown that newborns without risk factors for neurotoxicity, such as Rh incompatibility, sepsis, and lower admission weight, can tolerate bilirubin levels of 25 mg/dL or higher [6]. In the past three decades, several favorable biological properties of bilirubin, such as antioxidant, anti-inflammatory, antithrombotic, and lipid peroxidation inhibitory effects, have been demonstrated both in vivo and in vitro [7,8]. Gilbert’s syndrome is a condition of mild hyperbilirubinemia (serum bilirubin levels of 1 to 5 mg/dL) and is caused by a mutation in the gene promoter for *UGT1A1*, with marked impairment of bilirubin conjugation and excretion [9]. Most people with Gilbert’s syndrome are asymptomatic; however, the episodes of jaundice can be triggered by a wide range of circumstances such as exercise, fasting, and intercurrent illness [10]. Recent research has shown an association of a high bilirubin level with low risks of cardiovascular disease (CVD) and death in this population [11,12]. Furthermore, several epidemiological studies have also revealed that higher serum bilirubin concentrations in physiological ranges (1–5 mg/dL) were associated with decreased risk of hypertension, obesity, diabetes mellitus, the development and progression of chronic kidney disease (CKD), and all-cause mortality [13,14,15,16]. These results indicated the imperative role of bilirubin in human physiology.

**Figure 2:**
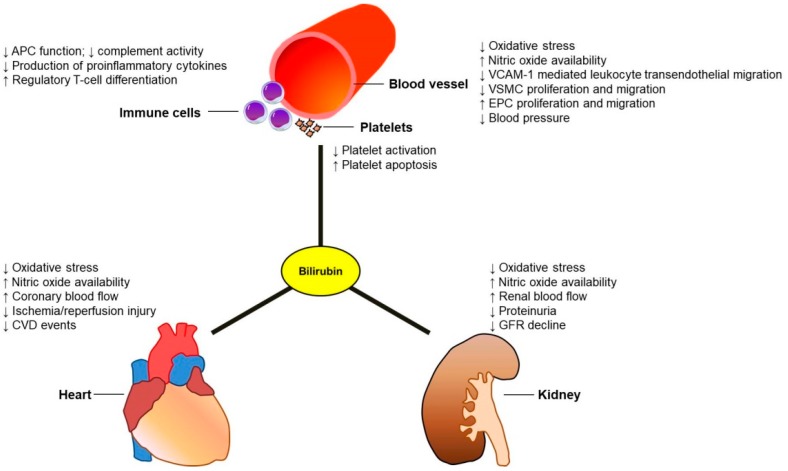
Cardiovascular and renal effects of bilirubin. In blood vessels, bilirubin can reduce oxidative stress and increase nitric oxide levels to maintain cardiovascular homeostasis. Bilirubin can also decrease the size of atherosclerotic plaques following vascular endothelial injury, through the suppression of VSMC proliferation and migration as well as improved EPC function. In addition, there is accumulating evidence that bilirubin has an antithrombotic effect by inhibiting platelet aggregation and promoting its apoptosis. Finally, bilirubin may exert its immunomodulatory effects by interacting with both the innate and adaptive responses. These findings support a potential role for bilirubin in protecting from the development and progression of both chronic kidney disease and cardiovascular disease. Abbreviations: APC, antigen-presenting cells; CVD, cardiovascular disease; EPC, endothelial progenitor cells; GFR, glomerular filtration rate; VCAM-1, vascular cell adhesion molecule 1; VSMC, vascular smooth muscle cells.

The prevalence of CKD is increasing worldwide, corresponding to an increased risk of premature death, especially from CVD. Despite progress in medical treatment over the past decades, clinical outcomes in patients with advanced CKD, particularly in those with end-stage renal disease (ESRD), are not significantly improved over time [17,18]. Therefore, development of promising novel therapeutic approaches to extend the lifespan of these patients is needed. The potential mechanisms of action of bilirubin in the prevention of the progression of CKD and renal transplant rejection have recently been discussed, and we refer the interested readers to these review papers for more information [19,20]. In this review, we described the physiological characteristics of bilirubin and how these factors may influence the clinical course and outcomes of CKD (Figure 2). We also proposed the potential therapeutic interventions aiming to induce hyperbilirubinemia that might be employed to improve clinical outcome and survival in these patients.

## 2. Potential Effects of Bilirubin against Cardiovascular Events in CKD Patients

### 2.1. Antioxidant Effects of Bilirubin

Renal disease is characterized by increased oxidative stress even in the early CKD [21]. The extent of oxidative damage to biological structures is most prominent in patients with ESRD, and longer dialysis vintage is associated with further elevation of markers of oxidative stress [22]. This could be a consequence of an increase in free radical production, a decrease in antioxidant defense, or both. Persistent oxidative damage can trigger the inflammatory process, leading to accelerate renal injury with progression to ESRD and increase the risk of CVD in CKD patients. Accordingly, molecules targeting oxidative stress could be a strategy for the treatment of CKD [23].

Bilirubin has potent antioxidant activities whether it is free or albumin-bound, and unconjugated or conjugated [24]. In 1987, Stocker et al. first reported that bilirubin, at micromolar concentrations, exhibited a powerful antioxidant that scavenged the peroxyl radicals, which is more efficient as compared to α-tocopherol. Therefore, it is regarded as a top antioxidant against lipid peroxidation [25]. Bilirubin also directly inhibited NADPH oxidase activity and suppressed superoxide generation in vascular endothelial cells and renal tubular cells [26]. Similarly, both endogenous and exogenous bilirubins have been shown to attenuate oxidative stress and renal dysfunction in animal studies. Oh et al. demonstrated that intraperitoneal administration of bilirubin markedly improved tubular injury and interstitial fibrosis via protection from oxidative stress and apoptosis in a rat model of cyclosporine nephropathy [27]. In another study, Fujii et al. demonstrated a protective effect of bilirubin against diabetic nephropathy in Gunn rats (UGT1A1-deficient hyperbilirubinemic) [28]. Streptozotocin-treated Gunn rats had less albuminuria and significantly reduced mesangial expansion accompanied by downregulation of NADPH oxidase when compared to streptozotocin-treated wild-type littermate controls [28].

The intracellular concentration of bilirubin is relatively low, but its antioxidant activities might be amplified tremendously by the bilirubin-biliverdin redox cycle, in which bilirubin is regenerated via biliverdin reductase [7,29]. Moreover, individuals with Gilbert’s syndrome also had higher circulating levels of thiol-containing antioxidants such as reduced glutathione (GSH) compared with controls, and the level of GSH was positively correlated with the bilirubin concentration [30]. These antioxidants may operate in a complementary way to form an integrated antioxidant system in the body [31]. Therefore, elevated bilirubin in vivo increased circulating antioxidant capacity and could inhibit accumulation of oxidative stress, which may have implications for the pathogenesis of CKD and its related cardiovascular complications.

### 2.2. Immunomodulatory and Anti-Inflammatory Effects of Bilirubin

Chronic low-grade inflammation is prevalent in patients with CKD. The causes of inflammation in CKD are multifactorial, including increased production and inadequate removal of pro-inflammatory cytokines, uremic toxins and oxidative stress, intercurrent infections, metabolic acidosis, and the dialysis procedure [32,33,34]. The process of persistent inflammation can lead to the progression of kidney disease, and development of anemia, protein energy wasting, insulin resistance and cardiovascular complications among patients with CKD [35,36,37]. A number of novel therapeutic strategies to reduce inflammation in CKD are currently being explored; however, progress in development of new anti-inflammatory drugs is hampered by the complexity of the molecular signaling network involved in inflammation and the essential characteristics of some of these signals for cell survival [38,39].

Bilirubin and factors affecting its metabolism such as HO can induce various immunomodulatory effects on the immune system. HO also has a diverse range of effects on the vasculature including the reduction of free radicals, relaxation of vascular tone, inhibition of smooth muscle cell growth as well as promotion of angiogenesis [40]. Unconjugated bilirubin at a physiologic level can cause impairment of antigen presentation in macrophages through modulating the expression of surface markers, such as MHC class II molecules, B7, as well as several different types of Fc receptors [41]. The classical complement pathway is also affected by bilirubin through interfering with C1 complex-immunoglobulins interaction [42]. In addition, Keshavan et al. demonstrated that bilirubin is able to suppress inflammatory responses in vivo by preventing leukocyte infiltration into target tissues through blocking vascular cell adhesion molecule-1 (VCAM-1) signaling in endothelial cells [43]. The authors further concluded that the inhibitory effects of bilirubin on transendothelial leukocyte migration were mediated by scavenging NADPH oxidase-generated reactive oxygen species (ROS) [43].

Apart from the innate immunity, bilirubin also has an important role in the adaptive immune system. In vitro, a decrease in mitogen-stimulated proliferation and production of proinflammatory cytokines, such as IL-2, IL-4, IL-10 and IFN-γ, has been reported in CD4^+^ T lymphocytes after incubation with bilirubin (50 to 150 μM) [41]. The beneficial effect of bilirubin was further confirmed in vivo by reduction of CD4^+^ T cells infiltration and proinflammatory cytokine expression in brain tissue when administration of bilirubin in experimental autoimmune encephalomyelitis, a rodent model of multiple sclerosis [41]. Moreover, some investigations have shown that bilirubin can induce tolerance to allografts in several animal models of allotransplantation [20]. In a mouse model of islet cell transplantation, recipients treated with bilirubin had better graft survival than vehicle-treated mice [44]. Recipient treatment with bilirubin induced expansion of Foxp3-positive regulatory T cells and suppressed expressions of proinflammatory and proapoptotic genes in the islet grafts [44]. Deetman et al. also found that circulating levels of bilirubin are inversely and independently associated with late graft failure in a cohort of kidney transplant recipients [45]. Therefore, cumulative evidence has shown that bilirubin acts as an immunomodulatory agent at concentrations higher than normal levels. Whether these important findings in the context of chronic inflammatory diseases are pertained to CKD still remains to be evaluated.

### 2.3. Antithrombotic Effects of Bilirubin

The risk of arterial and venous thromboembolism is increased in patients with CKD. In addition to traditional cardiovascular risk factors, both oxidative stress and inflammation can activate pathways contributing to thrombotic cardiovascular events in uremia [46,47]. Evaluation of vascular thrombosis should be undertaken in the early stages of CKD, as the prevalence of these complications begins to rise when microalbuminuria is present [48]. Moreover, platelets also have a role in the progression of CKD. Several cytokines and growth factors released from platelets were increased in some animal models of nephritis and can effectively influence the glomerular histological changes through expansion of the mesangial extracellular matrix with subsequent glomerular sclerosis [49,50]. However, current data on anticoagulant and antiplatelet therapy for slowing disease progression or to prevent the occurrence of vascular events in patients with CKD, particularly in those with ESRD, were conflicting or inconclusive [51,52,53,54].

Recent studies have shown that bilirubin can reduce the risk of thrombosis and CVD through direct and indirect mechanisms. As described in the previous section, the effects of bilirubin on lipid-lowering, anti-inflammatory, antioxidant, and endothelial homeostatic properties are inversely associated with CVD risk in individuals of different clinical conditions, including CKD [19]. In addition, there is accumulating evidence that bilirubin, at concentrations seen in Gilbert’s syndrome, may inhibit platelet aggregation in vitro by directly altering functions of several receptors on platelet surface, such as receptors for collagen and ADP [55]. These findings were confirmed by a subsequent human study, demonstrating that expression of P-selectin on platelets after ADP stimulation was reduced in people with Gilbert’s syndrome, as was platelet aggregation in the presence of collagen and arachidonic acid [56]. Moreover, NaveenKumar et al. revealed that unconjugated bilirubin induced platelet apoptosis in vitro via mitochondrial ROS-induced p38 and p53 activation in a dose-dependent manner [57]. These observations were further validated in vivo using a phenylhydrazine-induced hyperbilirubinemic rat model as well as hyperbilirubinemic human subjects, demonstrating an increase in oxidative stress and apoptotic markers in circulating platelets [57]. In conclusion, bilirubin may play an important role in regulating platelet activation, aggregation, and life span during the progress of atherosclerosis that deserve further consideration as targets for CKD patients at high risk for CV complications.

### 2.4. Lipid Lowering Effects of Bilirubin

CKD is associated with dyslipidemia which comprises of normal to increased low-density lipoprotein cholesterol (LDL-C) concentrations, low high-density lipoprotein cholesterol (HDL-C) levels, and elevated levels of triglycerides (TGs), and apolipoprotein B (Apo B) containing lipoproteins [58]. These abnormalities of lipid metabolism may contribute to the development and progression of CVD in patients with CKD. The results of the Study of Heart and Renal Protection (SHARP) trial have found that lowering LDL-cholesterol with simvastatin plus ezetimibe can decrease the incidence of major atherosclerotic events in patients with moderate-to-severe CKD [59]; however, statin use does not reduce cardiovascular events in patients undergoing hemodialysis [60,61], nor does statin treatment confer any protection against progression of kidney disease [62]. Therefore, further research for the most appropriate management options is needed in this patient population.

A few studies have shown that relatively higher levels of bilirubin may have a beneficial effect on lipid metabolism. An inverse relationship between serum levels of bilirubin and circulating lipids, such as TGs, total cholesterol, and LDL-C was observed [63,64]. The negative correlation is also consistent for very-low-density lipoprotein (VLDL), intermediate-density lipoprotein (IDL), Apo-B, and the Apo-B/Apo-A1 ratio, which are also significant risk factors for CVD [64]. There are several possible mechanisms contributing to these beneficial effects. Firstly, the aryl hydrocarbon receptor (AhR) is a ligand-activated transcription factor that regulates the expression of multiple target genes [65]. It was shown that bilirubin can activate the AhR signaling pathways and may thus modulate the expression of genes involved in cholesterol and lipid metabolism [66]. Secondly, bilirubin can also exert lipid-lowering effects in vitro and in vivo by directly binding to peroxisome proliferator-activated receptor α (PPARα) and increasing its transcriptional activity [67]. Using the humanized mouse model of Gilbert’s syndrome polymorphism, it was further shown that the activation of PPARα by bilirubin is through reducing the phosphorylation of this nuclear receptor at serine 73 [68]. Finally, transintestinal and biliary cholesterol secretion both contribute to the disposal of excess cholesterol from the body [69]. Recent studies have suggested that elevated levels of circulating unconjugated bilirubin in Gilbert’s syndrome may have a positive impact on intestinal cholesterol excretion [64]. Further basic and clinical research is needed to elucidate the complex interactions between bilirubin and lipid homeostasis that would offer novel therapeutic strategies in the management of dyslipidemia among patients with CKD.

### 2.5. Bilirubin and Maintenance of Vascular Integrity

The vascular endothelium is a highly differentiated cellular monolayer that forms an active barrier between the bloodstream and the underlying tissues. The endothelial cells exert a remarkable impact on vascular homeostasis via the balance between a variety of relaxing and contractile factors in response to physiological and pathological stimuli. Endothelial dysfunction in patients with CKD may result from several conditions including hypertension or dyslipidemia, as well as nontraditional risk factors such as free radicals and inflammation [70]. Dysfunction of the systemic vascular endothelium can lead to vascular remodeling, and consequently the development and progression of CVD. Moreover, renal endothelial injury and microvascular dysfunction also play a critical role in renal fibrosis and progression of CKD through parenchymal hypoxia, local inflammation, and the process of endothelial-to-mesenchymal transition (EndMT) [71,72]. Therefore, the endothelium may represent an important therapeutic target in CKD.

Several studies have demonstrated the benefits of elevated bilirubin concentrations in the maintenance of endothelial homeostasis in general populations and patients at high risk for vascular events. Gullu et al. investigated the relationship between serum bilirubin levels and coronary endothelial function in young adults without cardiovascular risk factors [73]. They found that elevated bilirubin concentrations are associated with preserved coronary flow reserve and decreased levels of hsCRP. Elevated serum bilirubin levels were also found to be associated with decreased carotid intima-media thickness and lower plaque burden in patients with familial and nonfamilial dyslipidemia [74]. These data indicate that bilirubin can protect against coronary microvascular dysfunction and progression of atherosclerosis by reducing inflammation and improving endothelium-dependent vasodilation.

As stated before, oxidative stress is well-known for its involvement in the pathogenesis of the endothelial dysfunction and atherosclerosis [75]. In 2012, Maruhashi et al. first reported that high levels of bilirubin are associated with a significant reduction in oxidative stress biomarkers and enhanced endothelium-dependent flow-mediated vasodilation in people with Gilbert’s syndrome [76]. Furthermore, a recent study has found that physiological concentrations of bilirubin could dose-dependently inhibit VCAM-1- and ICAM-1-mediated migration of monocytes across activated human endothelial cells by scavenging intracellular ROS [77]. These findings were validated in a murine model of atherosclerosis, in which it was shown that administration of bilirubin significantly prevented atherosclerotic plaque formation and reduced inflammatory cell infiltration in aortic root lesions [77]. Thus, the potent antioxidant effects of bilirubin may offer a significant protection against endothelial dysfunction and atherosclerosis in patients at risk of CVD.

Bilirubin also has a profound impact on nitric oxide (NO) homeostasis in endothelial cells [78]. NO is the most important endothelium-derived relaxing substance, which plays a critical role in the protection against the onset and progression of CVD in patients with kidney disease. The bioactivity of endothelium-derived NO is reduced by superoxide, a major ROS. The excessive production of superoxide can react with NO to form the powerful oxidant peroxynitrite (ONOO^−^), which may induce extensive oxidative DNA and protein damage [79]. Decreased intracellular superoxide level under conditions of hyperbilirubinemia is therefore expected to reduce ONOO^−^ formation and increase the local NO bioavailability [8]. In addition, bilirubin can preserve local concentrations of NO via direct scavenging activity of ONOO^−^ [80].

Endothelial dysfunction in response to inflammatory processes and oxidative stress could induce vascular smooth muscle cell (VSMC) migration and proliferation [81]. Aberrant proliferation and migration of VSMC to the intima have been closely linked to the development and progression of atherosclerotic CVD [82]. Ollinger et al. firstly revealed that neointima formation was significantly reduced following carotid artery balloon injury in Gunn rats than in controls [83]. Using the same experimental model of vascular damage, Peyton et al. also found that local perivascular administration of bilirubin could attenuate neointima hyperplasia in wild-type rats [84]. These authors further reported that bilirubin exerted a dose-dependent anti-proliferative activity on VSMCs in vitro [84]. Bilirubin induced cell cycle arrest in the G0/G1 phase, which was mediated through inhibition of the mitogen-activated protein kinase (MAPK) signaling pathway and reduced phosphorylation of retinoblastoma protein [83]. Moreover, bilirubin also induces Ca^2+^ influx and calpain II activation that leads to increased proteolytic cleavage of YY1, an important transcription factor that regulates cell cycle progression in VSMCs [85].

Endothelial progenitor cells (EPCs) contribute to endothelial repair and angiogenesis after vascular injury [86]. Altered function and decreased number of circulating EPCs in patients with CKD have been well established and are recognized as key factors involved in the pathogenesis of endothelial dysfunction and CV complications [87]. A recent study by Jabarpour et al. found that circulating EPCs from infants with hyperbilirubinemia exhibited greater proliferative and migratory capacity as compared with EPCs from those with normal bilirubin levels [88]. Additionally, conditioned medium from EPCs isolated from hyperbilirubinemic infants could significantly increase levels of VEGF, IL-10, and p-ERK/ERK in the wound tissues and improve wound healing in the experimental animals [88]. Using in vitro functional assays and an in vivo murine hind-limb ischemia model, Ikeda et al. further demonstrated that bilirubin may directly promote angiogenesis through activation of Akt/eNOS signaling in endothelial cells [89].

The abovementioned studies extend the understanding of the role of bilirubin in the regulation of endothelial integrity; however, it remains to be validated whether elevated serum bilirubin levels, within the physiological range, can improve endothelial function and thus reduce cardiovascular morbidity and mortality in individuals with CKD.

### 2.6. Other Aspects of the Protective Action of Bilirubin

There are many other possible mechanisms how bilirubin might exert its protective action in patients with CKD, including the beneficial role of bilirubin on blood pressure [19], its antifibrotic effects [90], and the potential anti-obesity and anti-diabetic effects via improving insulin sensitivity and glucose homeostasis [91,92,93]. In addition to the above-mentioned intracellular mechanisms, bilirubin may inhibit the phosphorylation of several types of protein kinases, such as protein kinase C and MAPK cascades, leading to the modulation of intracellular signaling pathways in VSMCs [83]. Bilirubin is also a potent inducer of apoptosis with antiproliferative activity by promoting the expression of p53 in injured blood vessels, indicating the importance of bilirubin on the cell cycle [84]. While oxidative stress and inflammation are main determinants of CKD progression, nuclear factor erythroid-2-related factor-2 (Nrf2) confers protection against kidney damage by inducing antioxidant and detoxification responses to oxidative stress [94]. Recent studies have demonstrated that bilirubin is a potential endogenous compound that activate Nrf2 pathway under conditions of oxidative stress [95,96].

CKD is characterized by immune dysregulation, inflammatory activation, and metabolic and nutritional disturbances, which are all linked to dysbiotic gut microbiota [97]. As mentioned earlier, conjugated bilirubin is excreted in the bile and converted by intestinal microbiota to urobilinogens [98]. Urobilinogen is further processed to produce stercobilin, which gives feces their brown color, or reabsorbed from the gut. It is unknown whether lower serum levels of bilirubin in patients with CKD are due to changes in the gut microbial composition. Further investigation is needed to understand this process in humans and experimental animals.

## 3. Role of Bilirubin in Cardioprotection in CKD Patients at Risk of CVD

### 3.1. Evidence Supporting the Beneficial Effects of Bilirubin on CVD

Apart from the above experimental studies, clinical research also found a similar relationship existed between bilirubin levels and the risk of incident CKD, progression of CKD to ESRD and CVD mortality. In a prospective cohort study, 2784 Japanese subjects without CKD at baseline, defined as estimated glomerular filtration rate (eGFR) <60 mL/min/1.73 m^2^, were followed up for incidence of CKD over a period of 7.7 years [14]. There were 601 incident cases of CKD during this period. Results indicated that low serum total bilirubin concentration could be a predictor for the development of CKD after adjustment for the known risk factors. Serum bilirubin levels were also associated with the progression of kidney disease in patients with established CKD. A prospective cohort study of 279 people with stages 3–5 CKD and median follow-up period of 21 months demonstrated that the risk of poor renal outcome for each 0.1 mg/dL increase in bilirubin level was significantly reduced (hazard ratio (HR), 0.73; 95% confidence interval (CI), 0.60–0.87) after accounting for other potential confounders [99].

There are different causes of CKD, the most common being type 2 diabetes. Of the adult individuals with type 2 diabetes, the overall prevalence of nephropathy ranged from 34.5% to 42.3% [100]. Kidney involvement increases morbidity and mortality in this group of patients. Although several studies have demonstrated the association between circulating bilirubin and type 2 diabetes, the exact nature of the relationship remains unclear [101,102]. In a Mendelian randomization study, Abbasi et al. first reported a potential causal association between elevated total bilirubin levels and decreased risk of type 2 diabetes that is likely free of potential confounders [13]. Further studies are warranted to confirm this finding. Serum bilirubin level also has a significant impact on the onset and progression of diabetic nephropathy. In patients with type 2 diabetes and normoalbuminuria, Okada et al. reported a lower serum bilirubin level to be a risk factor for the development of albuminuria [103]. Moreover, Riphagen et al. found an independent inverse association of bilirubin levels with progression of diabetic nephropathy in a post hoc analysis from the RENAAL study and Irbesartan Diabetic Nephropathy Trial (IDNT) [104].

CVD is the leading cause of death among patients with ESRD. The association between serum bilirubin and long-term outcomes has been studied in our previous research involving 661 chronic hemodialysis patients followed up over a period of 12 years [105]. After adjusting for potential confounders, individuals with bilirubin in the upper versus the lower tertile had hazard ratios of 0.32 (0.21 to 0.48) for cardiovascular events (CVEs), and 0.48 (0.34 to 0.67) for all-cause mortality. Moreover, each 0.1 mg/dL increase in serum bilirubin decreased CVEs and all-cause mortality by 9% and 10%, respectively. In another cross-sectional study, Fukui and colleagues also found that a high serum bilirubin concentration is associated with lower incidence of CVEs among diabetic patients undergoing hemodialysis (odds ratio (OR), 0.192; 95% CI, 0.037–0.989; *P* = 0.0484) [106]. These potential benefits of bilirubin seen in ESRD patients under hemodialysis may be ascribed to its lipid-lowering and anti-inflammatory activity [107].

Recently, several studies have been conducted to determine the impact of bilirubin on longevity. Zelenka et al. demonstrated that bilirubin potentially reduces both mitochondrial and cytosolic ROS levels in a dose-dependent manner in human embryonic kidney cells and rat primary fibroblasts [108]. This effect is associated with decreased visceral fat deposition, inflammatory status, markers of cellular senescence, and mitochondrial dysfunction in aged hyperbilirubinemic Gunn rats. Tosevska et al. also showed that individuals with Gilbert’s syndrome have significantly longer telomeres compared with matched healthy controls [109]. This difference appears to be more pronounced with age, suggesting a slower telomere shortening rate in people chronically exposed to high levels of bilirubin. Finally, Chmielewski et al. revealed a statistically significant trend toward higher levels of bilirubin in men who had the highest age at death, which suggests that hyperbilirubinemia is associated with longer life span among older men [110]. However, the beneficial effect of bilirubin on longevity is not observed in older women. Therefore, further studies are needed to elucidate the underlying mechanisms of the relationship between serum bilirubin concentrations and longevity in the elderly of both genders.

### 3.2. Negative or Inconclusive Results

Although most clinical research has suggested a beneficial effect of bilirubin in the prevention of kidney disease and CVD, however, there are several investigations indicating a negative association between bilirubin and clinical outcomes. Wang et al. reported a significant inverse association between bilirubin levels and progression of CKD in never smokers, but not in ever smokers [111]. One limitation of this study is that the smoking status was not assessed in the follow-up periods. An assessment in continually smoking status is recommended for a better estimate of its effect. Su et al. conducted a retrospective nationwide cohort study that included 47,650 hemodialysis patients, using the Taiwan Renal Registry Data System (TWRDS) database from 2005 to 2012 [112]. These data indicated that total bilirubin is an independent risk factor of all-cause mortality for patients undergoing long-term hemodialysis. Nonetheless, the ratio between conjugated and unconjugated bilirubin was not determined in this study. Therefore, it is hard to know whether an elevated bilirubin in this study is caused by liver disease, hemolytic anemia or a benign condition such as Gilbert’s syndrome [112].

In addition to CKD, oxidative stress also plays an important role in acute kidney injury (AKI) [113]. Several kidney-specific biomarkers have been tested to improve early and accurate detection of AKI in clinical practice [114,115,116]. Although bilirubin has antioxidant and anti-inflammatory activity, the nature of the relationship between serum bilirubin and AKI is unclear. van Slambrouck et al. conducted a clinicopathologic study of 44 jaundiced patients at the University of Chicago [117]. The authors found that AKI is common in patients with severe liver dysfunction, such as liver cirrhosis. Moreover, patients with higher levels of serum total bilirubin have a greater risk of AKI compared to those who have lower bilirubin concentrations (26.2 vs. 15.1 mg/dL, *P* = 0.001). Renal histology from these patients showed marked tubular injury with epithelial cell necrosis, loss of brush border, and extensive bile cast formation in tubules. The association between higher bilirubin levels and the development of AKI in liver cirrhosis might be partially explained by direct cytotoxicity and tubular obstruction mediated via bile casts [117,118]. Therefore, evaluation of the impact of bilirubin on renal function must be accompanied by an assessment of liver function to avoid confounding by ill health.

Other negative or inconclusive results of bilirubin on study endpoints were observed mainly in patients with multiple comorbidities such as hemorrheological disorders, infectious diseases, and decompensated heart failure, which confound the effect of bilirubin on prognosis and should be interpreted with caution [19].

### 3.3. Gene Polymorphisms Involved in Bilirubin Metabolism and Their Relationship with CKD Progression and Cardiovascular Mortality—Focused on *UGT1A1* and *HMOX1* Gene Polymorphisms

Growing literature has shown that serum levels of bilirubin are highly heritable in humans. [119]. The genetic factors under investigation include genetic defects affecting the lifespan of red blood cells as well as polymorphisms involved in bilirubin metabolism, such as *HMOX1*, *SLCO1B1* (solute carrier organic anion transporter family member 1B1), and *UGT1A1* genes [120]. The role of these genetic modifiers on clinical outcomes in CKD patients at higher risk for cardiovascular complications has become a favorite topic of analysis in the field of translational research [105,121,122]. The *UGT1A1* and *HMOX1* polymorphisms have been widely studied during the past few years.

In humans, a (GT)_n_ dinucleotide repeat in the *HMOX1* gene promoter shows length polymorphism and could modulate the level of gene expression [123]. The long polymorphism (L allele), defined as the number of repeats greater than 25 to 27 in this promoter sequence, is associated with a decrease in HO-1 protein expression and enzyme activity, and therefore reduces the formation of bilirubin, which may elevate the risk of CKD, progression of kidney disease, and cardiovascular mortality [124]. We have previously reported a 10.2-year follow-up in a cohort of 386 patients with coronary artery disease for the potential detrimental effects of the L allele on serum bilirubin levels and renal function decline [125]. Baseline serum bilirubin level was significantly lower in patients with L/L genotype compared to those homozygous for the S allele. At the end of study, individuals with the L/L genotype had a greater risk of developing renal endpoints (doubling of serum creatinine and/or ESRD requiring dialysis), CVEs, and mortality than carriers of the short (S) allele. In another study, we analyzed whether the length polymorphism of the (GT)_n_ in the *HMOX1* promoter was an independent predictor of CVEs and all-cause mortality in 1080 chronic hemodialysis patients [122]. The patients with the L/L genotype had significantly higher baseline levels of high-sensitivity C-reactive protein and malondialdehyde than those with the S/S or S/L genotypes. After multivariate adjustment, the homozygous L/L patients had a significantly higher risk for death and cardiovascular complications than the S allele carriers. The associations between *HMOX1* promoter polymorphisms and clinical outcomes were also observed among patients with other forms of kidney disease, such as renal transplantation, diabetic kidney disease and sickle cell nephropathy [125,126,127].

Of the genes implicated in bilirubin metabolism, *UGT1A1* has been the most widely studied because of its essential role to catalyze bilirubin glucuronidation that contributes substantially to bilirubin elimination in humans. A common cause of defective UGT1A1 enzyme activity results from a TA insertion in the TATAA box in the promoter region of the *UGT1A1* gene, resulting in a (TA)_7_TAA allele (UGT1A1*28) instead of the normal (TA)_6_TAA allele [128]. Persons with homozygous for UGT1A1*28 (7/7) had lower UGT1A1 enzyme activity and subsequent higher levels of serum bilirubin than individuals who are heterozygotes (6/7) or those homozygous for the wild-type allele (6/6) [129]. To date, many studies have investigated the association of the UGT1A1*28 polymorphism with CVD in different clinical settings [78]. However, some research examining this association has shown inconsistent results [130,131]. We have explored the association between the UGT1A1*28 allele and serum bilirubin and the effect of this genetic variant on adverse outcomes in a cohort of long-term hemodialysis patients [105]. In a median follow-up of 54 months, patients of homozygous for UGT1A1*28 had significantly higher bilirubin concentrations than those with 6/7 and 6/6 genotypes. Moreover, individuals with the 7/7 genotype had only one-tenth the risk for CVD and one-fourth the risk for all-cause mortality as the carriers of the wild-type allele. Further studies are needed to establish a definite causal relationship among *UGT1A1* gene polymorphisms, bilirubin levels and CVEs in patients with CKD.

## 4. Interventions to Modulate Bilirubin Levels as a New Therapeutic Strategy for Patients with CKD and CVD

The potential protective effect of bilirubin could make it a valuable strategy for pharmacologic intervention to prevent the development, progression, and cardiovascular complications of CKD. Dekker et al. demonstrated that it is feasible to prepare and apply albumin-bound bilirubin for parenteral human use under Good Manufacturing Practice (GMP) standards [132]. Following a single bolus infusion, it appeared to be safe to parenterally administer bilirubin up to plasma concentrations of 86 µM (5.03 mg/dL) during the 2-week observation period. Exploratory pharmacokinetic data had also suggested that there was a predictable dose–concentration relationship for this formulation of bilirubin solutions. This study may open the gate to the therapeutic use of bilirubin for future clinical and translational research.

Systemic elevation of bilirubin can be achieved by induction of HO, the enzyme involved in the key step of bilirubin formation [1]. Several natural and synthetic compounds have been shown to possess antioxidant and anti-inflammatory activities through the induction of HO protein expression and HO activity, such as statins, aspirin and curcumin [133]. A recent study reported that direct intraperitoneal administration of bilirubin, as well as induction of HO-1 by hemin, augmented endothelium-dependent relaxations and increased phosphorylation of Akt and eNOS in db/db mouse aortas [134]. The vasoprotective effect of hemin but not bilirubin was inhibited by silencing biliverdin reductase expression. These results indicated that HO-1—induced preservation of endothelial function is most likely mediated by bilirubin.

There are two isoforms of biliverdin reductase, biliverdin reductase A (BLVRA), the major isoenzyme present in adult tissues, and biliverdin reductase B (BLVRB), expressed predominantly in the developing fetal liver [135]. In addition to its reductase activity, BLVRA also has other unique domains which allow it to interact with different signal transduction pathways, such as MAPK, protein kinase C, and insulin receptor kinase (IRK) [136]. Several studies have demonstrated that the C-terminal peptide of the human BLVRA protein, KYCCSRK, can enhance IRK activity and improve glucose uptake in several types of cells, and in wild-type and diabetic Ob/Ob mice [137]. Recent evidence suggests that BLVRA might have relevant pathophysiological implications in both heart and kidney disease [138]. Therefore, further investigations are required to determine the potential for the development of BLVRA based peptides for the treatment of CKD and CVD.

Inhibition of UGT1A1-mediated bilirubin glucuronidation is another strategy to increase serum levels of unconjugated bilirubin. Drugs known to inhibit UGT1A1 include atazanavir, gemfibrozil and probenecid [139]. In fact, LaFleur et al. found that atazanavir-containing regimens were associated with a significantly lower risk for both myocardial infarction and stroke in treatment-naïve patients with HIV infection compared with non-atazanavir-containing regimens [140]. Moreover, atazanavir has also been demonstrated to improve endothelial function in patients with type 2 diabetes mellitus, possibly due to its effects on serum bilirubin levels [141]. However, UGT1A1 inhibition may not only bring risks of drug–drug interactions, but also result in drug-induced liver injury [142]. These may cause drug-related side effects, which can lead to serious adverse events if not promptly identified.

## 5. Conclusions

In conclusion, animal experiments and clinical observations have provided some compelling evidence to support a role for bilirubin in protecting from the development and progression of both CKD and CVD. Multiple mechanisms are proposed to explain the beneficial effect of bilirubin in CKD patients at high risk of atherosclerotic disease, including antioxidant, anti-inflammatory and endothelium-protective effects, indicating that it could serve as a potentially therapeutic target for slowing the progression of CKD and CVD. While direct administration of bilirubin has been evaluated with some success in several animal experiments for a variety of diseases, such as obesity [91], diabetes mellitus [134] and acute myocardial injury [143], the use of bilirubin in humans requires additional preclinical studies. Moreover, recent investigations have also found mild hyperbilirubinemia that can be induced by either enhancement of HO activity or partial inhibition of UGT1A1, which has therapeutic potential for delaying CKD progression and improving cardiovascular outcomes in this patient population.

## References

[1] Rochette L., Zeller M., Cottin Y., Vergely C. (2018). Redox Functions of Heme Oxygenase-1 and Biliverdin Reductase in Diabetes. Trends Endocrinol. Metab..

[2] O’Brien L., Hosick P.A., John K., Stec D.E., Hinds T.D. (2015). Biliverdin reductase isozymes in metabolism. Trends Endocrinol. Metab..

[3] Beutler E., Gelbart T., Demina A. (1998). Racial variability in the UDP-glucuronosyltransferase 1 (UGT1A1) promoter: A balanced polymorphism for regulation of bilirubin metabolism?. Proc. Natl. Acad. Sci. USA.

[4] Keppler D. (2014). The roles of MRP2, MRP3, OATP1B1, and OATP1B3 in conjugated hyperbilirubinemia. Drug Metab. Dispos..

[5] Hinds T.D., Burns K.A., Hosick P.A., McBeth L., Nestor-Kalinoski A., Drummond H.A., AlAmodi A.A., Hankins M.W., Vanden Heuvel J.P., Stec D.E. (2016). Biliverdin Reductase A Attenuates Hepatic Steatosis by Inhibition of Glycogen Synthase Kinase (GSK) 3beta Phosphorylation of Serine 73 of Peroxisome Proliferator-activated Receptor (PPAR) alpha. J. Biol. Chem..

[6] Gamaleldin R., Iskander I., Seoud I., Aboraya H., Aravkin A., Sampson P.D., Wennberg R.P. (2011). Risk factors for neurotoxicity in newborns with severe neonatal hyperbilirubinemia. Pediatrics.

[7] Gazzin S., Vitek L., Watchko J., Shapiro S.M., Tiribelli C. (2016). A Novel Perspective on the Biology of Bilirubin in Health and Disease. Trends Mol. Med..

[8] Bulmer A.C., Bakrania B., Du Toit E.F., Boon A.C., Clark P.J., Powell L.W., Wagner K.H., Headrick J.P. (2018). Bilirubin acts as a multipotent guardian of cardiovascular integrity: More than just a radical idea. Am. J. Physiol. Heart Circ. Physiol..

[9] Wagner K.H., Shiels R.G., Lang C.A., Seyed Khoei N., Bulmer A.C. (2018). Diagnostic criteria and contributors to Gilbert’s syndrome. Crit. Rev. Clin. Lab. Sci..

[10] Powell L.W., Hemingway E., Billing B.H., Sherlock S. (1967). Idiopathic unconjugated hyperbilirubinemia (Gilbert’s syndrome). A study of 42 families. N. Engl. J. Med..

[11] Vitek L., Jirsa M., Brodanova M., Kalab M., Marecek Z., Danzig V., Novotny L., Kotal P. (2002). Gilbert syndrome and ischemic heart disease: A protective effect of elevated bilirubin levels. Atherosclerosis.

[12] Marconi V.C., Duncan M.S., So-Armah K., Re V.L., Lim J.K., Butt A.A., Goetz M.B., Rodriguez-Barradas M.C., Alcorn C.W., Lennox J. (2018). Bilirubin Is Inversely Associated With Cardiovascular Disease Among HIV-Positive and HIV-Negative Individuals in VACS (Veterans Aging Cohort Study). J. Am. Heart Assoc..

[13] Abbasi A., Deetman P.E., Corpeleijn E., Gansevoort R.T., Gans R.O., Hillege H.L., van der Harst P., Stolk R.P., Navis G., Alizadeh B.Z. (2015). Bilirubin as a potential causal factor in type 2 diabetes risk: A Mendelian randomization study. Diabetes.

[14] Tanaka M., Fukui M., Okada H., Senmaru T., Asano M., Akabame S., Yamazaki M., Tomiyasu K., Oda Y., Hasegawa G. (2014). Low serum bilirubin concentration is a predictor of chronic kidney disease. Atherosclerosis.

[15] Wagner K.H., Wallner M., Molzer C., Gazzin S., Bulmer A.C., Tiribelli C., Vitek L. (2015). Looking to the horizon: The role of bilirubin in the development and prevention of age-related chronic diseases. Clin. Sci..

[16] Huang S.S., Chan W.L., Leu H.B., Huang P.H., Lin S.J., Chen J.W. (2015). Serum bilirubin levels predict future development of metabolic syndrome in healthy middle-aged nonsmoking men. Am. J. Med..

[17] Levin A., Tonelli M., Bonventre J., Coresh J., Donner J.A., Fogo A.B., Fox C.S., Gansevoort R.T., Heerspink H.J.L., Jardine M. (2017). Global kidney health 2017 and beyond: A roadmap for closing gaps in care, research, and policy. Lancet.

[18] Stenvinkel P., Painer J., Kuro O.M., Lanaspa M., Arnold W., Ruf T., Shiels P.G., Johnson R.J. (2018). Novel treatment strategies for chronic kidney disease: Insights from the animal kingdom. Nat. Rev. Nephrol..

[19] Boon A.C., Bulmer A.C., Coombes J.S., Fassett R.G. (2014). Circulating bilirubin and defense against kidney disease and cardiovascular mortality: Mechanisms contributing to protection in clinical investigations. Am. J. Physiol. Renal Physiol..

[20] Sundararaghavan V.L., Binepal S., Stec D.E., Sindhwani P., Hinds T.D. (2018). Bilirubin, a new therapeutic for kidney transplant?. Transplant. Rev. (Orlando).

[21] Duni A., Liakopoulos V. (2017). Chronic Kidney Disease and Disproportionally Increased Cardiovascular Damage: Does Oxidative Stress Explain the Burden?. Oxid. Med. Cell. Longev..

[22] Xu H., Watanabe M., Qureshi A.R., Heimburger O., Barany P., Anderstam B., Eriksson M., Stenvinkel P., Lindholm B. (2015). Oxidative DNA damage and mortality in hemodialysis and peritoneal dialysis patients. Perit. Dial. Int..

[23] Signorini L., Granata S., Lupo A., Zaza G. (2017). Naturally Occurring Compounds: New Potential Weapons against Oxidative Stress in Chronic Kidney Disease. Int. J. Mol. Sci..

[24] Mayer M. (2000). Association of serum bilirubin concentration with risk of coronary artery disease. Clin. Chem..

[25] Stocker R., Yamamoto Y., McDonagh A.F., Glazer A.N., Ames B.N. (1987). Bilirubin is an antioxidant of possible physiological importance. Science.

[26] Ratliff B.B., Abdulmahdi W., Pawar R., Wolin M.S. (2016). Oxidant Mechanisms in Renal Injury and Disease. Antioxid. Redox Signal..

[27] Oh S.W., Lee E.S., Kim S., Na K.Y., Chae D.W., Kim S., Chin H.J. (2013). Bilirubin attenuates the renal tubular injury by inhibition of oxidative stress and apoptosis. BMC Nephrol.

[28] Fujii M., Inoguchi T., Sasaki S., Maeda Y., Zheng J., Kobayashi K., Takayanagi R. (2010). Bilirubin and biliverdin protect rodents against diabetic nephropathy by downregulating NAD(P)H oxidase. Kidney Int..

[29] Vitek L. (2017). Bilirubin and atherosclerotic diseases. Physiol. Res..

[30] Boon A.C., Hawkins C.L., Bisht K., Coombes J.S., Bakrania B., Wagner K.H., Bulmer A.C. (2012). Reduced circulating oxidized LDL is associated with hypocholesterolemia and enhanced thiol status in Gilbert syndrome. Free Radic. Biol. Med..

[31] Sedlak T.W., Saleh M., Higginson D.S., Paul B.D., Juluri K.R., Snyder S.H. (2009). Bilirubin and glutathione have complementary antioxidant and cytoprotective roles. Proc. Natl. Acad. Sci. USA.

[32] Hung S.C., Kuo K.L., Wu C.C., Tarng D.C. (2017). Indoxyl Sulfate: A Novel Cardiovascular Risk Factor in Chronic Kidney Disease. J. Am. Heart Assoc..

[33] Jha J.C., Ho F., Dan C., Jandeleit-Dahm K. (2018). A causal link between oxidative stress and inflammation in cardiovascular and renal complications of diabetes. Clin. Sci..

[34] Kraut J.A., Madias N.E. (2016). Metabolic Acidosis of CKD: An Update. Am. J. Kidney Dis..

[35] Spoto B., Pisano A., Zoccali C. (2016). Insulin resistance in chronic kidney disease: A systematic review. Am. J. Physiol. Renal Physiol..

[36] Bhatti N.K., Karimi Galougahi K., Paz Y., Nazif T., Moses J.W., Leon M.B., Stone G.W., Kirtane A.J., Karmpaliotis D., Bokhari S. (2016). Diagnosis and Management of Cardiovascular Disease in Advanced and End-Stage Renal Disease. J. Am. Heart Assoc..

[37] Tsai M.T., Hu F.H., Lien T.J., Chen P.J., Huang T.P., Tarng D.C. (2014). Interaction between geriatric nutritional risk index and decoy receptor 3 predicts mortality in chronic hemodialysis patients. Am. J. Nephrol..

[38] Machowska A., Carrero J.J., Lindholm B., Stenvinkel P. (2016). Therapeutics targeting persistent inflammation in chronic kidney disease. Transl. Res..

[39] Sumida K., Molnar M.Z., Potukuchi P.K., Hassan F., Thomas F., Yamagata K., Kalantar-Zadeh K., Kovesdy C.P. (2018). Treatment of rheumatoid arthritis with biologic agents lowers the risk of incident chronic kidney disease. Kidney Int..

[40] Ayer A., Zarjou A., Agarwal A., Stocker R. (2016). Heme Oxygenases in Cardiovascular Health and Disease. Physiol. Rev..

[41] Liu Y., Li P., Lu J., Xiong W., Oger J., Tetzlaff W., Cynader M. (2008). Bilirubin possesses powerful immunomodulatory activity and suppresses experimental autoimmune encephalomyelitis. J. Immunol..

[42] Basiglio C.L., Arriaga S.M., Pelusa H.F., Almara A.M., Roma M.G., Mottino A.D. (2007). Protective role of unconjugated bilirubin on complement-mediated hepatocytolysis. Biochim. Biophys. Acta.

[43] Keshavan P., Deem T.L., Schwemberger S.J., Babcock G.F., Cook-Mills J.M., Zucker S.D. (2005). Unconjugated bilirubin inhibits VCAM-1-mediated transendothelial leukocyte migration. J. Immunol..

[44] Rocuts F., Zhang X., Yan J., Yue Y., Thomas M., Bach F.H., Czismadia E., Wang H. (2010). Bilirubin promotes de novo generation of T regulatory cells. Cell Transplant..

[45] Deetman P.E., Zelle D.M., Homan van der Heide J.J., Navis G.J., Gans R.O., Bakker S.J. (2012). Plasma bilirubin and late graft failure in renal transplant recipients. Transpl. Int..

[46] Ribic C., Crowther M. (2016). Thrombosis and anticoagulation in the setting of renal or liver disease. Hematology Am. Soc. Hematol. Educ. Program.

[47] Kolachalama V.B., Shashar M., Alousi F., Shivanna S., Rijal K., Belghasem M.E. (2018). Uremic Solute-Aryl Hydrocarbon Receptor-Tissue Factor Axis Associates with Thrombosis after Vascular Injury in Humans. J. Am. Soc. Nephrol..

[48] Ocak G., Verduijn M., Vossen C.Y., Lijfering W.M., Dekker F.W., Rosendaal F.R., Gansevoort R.T., Mahmoodi B.K. (2010). Chronic kidney disease stages 1-3 increase the risk of venous thrombosis. J. Thromb. Haemost..

[49] Kitching A.R., Hutton H.L. (2016). The Players: Cells Involved in Glomerular Disease. Clin. J. Am. Soc. Nephrol..

[50] Lambert M.P. (2016). Platelets in liver and renal disease. Hematol. Am. Soc. Hematol. Educ. Program.

[51] Donadio J.V., Anderson C.F., Mitchell J.C., Holley K.E., Ilstrup D.M., Fuster V., Chesebro J.H. (1984). Membranoproliferative glomerulonephritis. A prospective clinical trial of platelet-inhibitor therapy. N. Engl. J. Med..

[52] Lozano I., Rondan J., Vegas J.M., Segovia E. (2018). Chronic Kidney Disease and Antiplatelet Therapy: A Worrying Gap Between Evidence Based Medicine and Clinical Practice. JACC Cardiovasc. Interv..

[53] Lee K.H., Li S.Y., Liu J.S., Huang C.T., Chen Y.Y., Lin Y.P., Hsu C.C., Tarng D.C. (2017). Association of warfarin with congestive heart failure and peripheral artery occlusive disease in hemodialysis patients with atrial fibrillation. J. Chin. Med. Assoc..

[54] Tsai M.T., Chen Y.Y., Chang W.J., Li S.Y. (2018). Warfarin accelerated vascular calcification and worsened cardiac dysfunction in remnant kidney mice. J. Chin. Med. Assoc..

[55] Kundur A.R., Singh I., Bulmer A.C. (2015). Bilirubin, platelet activation and heart disease: A missing link to cardiovascular protection in Gilbert’s syndrome?. Atherosclerosis.

[56] Kundur A.R., Santhakumar A.B. (2017). Mildly elevated unconjugated bilirubin is associated with reduced platelet activation-related thrombogenesis and inflammation in Gilbert’s syndrome. Platelets.

[57] NaveenKumar S.K., Thushara R.M., Sundaram M.S., Hemshekhar M., Paul M., Thirunavukkarasu C., Basappa, Nagaraju G., Raghavan S.C., Girish K.S. (2015). Unconjugated Bilirubin exerts Pro-Apoptotic Effect on Platelets via p38-MAPK activation. Sci. Rep..

[58] Bulbul M.C., Dagel T., Afsar B., Ulusu N.N., Kuwabara M., Covic A., Kanbay M. (2018). Disorders of Lipid Metabolism in Chronic Kidney Disease. Blood Purif..

[59] Baigent C., Landray M.J., Reith C., Emberson J., Wheeler D.C., Tomson C., Wanner C., Krane V., Cass A., Craig J. (2011). The effects of lowering LDL cholesterol with simvastatin plus ezetimibe in patients with chronic kidney disease (Study of Heart and Renal Protection): A randomised placebo-controlled trial. Lancet.

[60] Wanner C., Krane V., Marz W., Olschewski M., Mann J.F., Ruf G., Ritz E. (2005). Atorvastatin in patients with type 2 diabetes mellitus undergoing hemodialysis. N. Engl. J. Med..

[61] Fellstrom B.C., Jardine A.G., Schmieder R.E., Holdaas H., Bannister K., Beutler J., Chae D.W., Chevaile A., Cobbe S.M., Gronhagen-Riska C. (2009). Rosuvastatin and cardiovascular events in patients undergoing hemodialysis. N. Engl. J. Med..

[62] Haynes R., Lewis D., Emberson J., Reith C., Agodoa L., Cass A., Craig J.C., de Zeeuw D., Feldt-Rasmussen B., Fellstrom B. (2014). Effects of lowering LDL cholesterol on progression of kidney disease. J. Am. Soc. Nephrol..

[63] Oda E. (2014). A decrease in total bilirubin predicted hyper-LDL cholesterolemia in a health screening population. Atherosclerosis.

[64] Bulmer A.C., Verkade H.J., Wagner K.H. (2013). Bilirubin and beyond: A review of lipid status in Gilbert’s syndrome and its relevance to cardiovascular disease protection. Prog. Lipid Res..

[65] Dietrich C. (2016). Antioxidant Functions of the Aryl Hydrocarbon Receptor. Stem Cells Int..

[66] Phelan D., Winter G.M., Rogers W.J., Lam J.C., Denison M.S. (1998). Activation of the Ah receptor signal transduction pathway by bilirubin and biliverdin. Arch. Biochem. Biophys..

[67] Stec D.E., John K., Trabbic C.J., Luniwal A., Hankins M.W., Baum J., Hinds T.D. (2016). Bilirubin Binding to PPARalpha Inhibits Lipid Accumulation. PLoS ONE.

[68] Hinds T.D., Hosick P.A., Chen S., Tukey R.H., Hankins M.W., Nestor-Kalinoski A., Stec D.E. (2017). Mice with hyperbilirubinemia due to Gilbert’s syndrome polymorphism are resistant to hepatic steatosis by decreased serine 73 phosphorylation of PPARalpha. Am. J. Physiol. Endocrinol. Metab..

[69] De Boer J.F., Schonewille M., Dikkers A., Koehorst M., Havinga R., Kuipers F., Tietge U.J., Groen A.K. (2017). Transintestinal and Biliary Cholesterol Secretion Both Contribute to Macrophage Reverse Cholesterol Transport in Rats-Brief Report. Arterioscler. Thromb. Vasc. Biol..

[70] Martens C.R., Kirkman D.L., Edwards D.G. (2016). The Vascular Endothelium in Chronic Kidney Disease: A Novel Target for Aerobic Exercise. Exerc. Sport Sci. Rev..

[71] Perry H.M., Okusa M.D. (2016). Endothelial Dysfunction in Renal Interstitial Fibrosis. Nephron.

[72] Lipphardt M., Song J.W., Matsumoto K., Dadafarin S., Dihazi H., Muller G., Goligorsky M.S. (2017). The third path of tubulointerstitial fibrosis: Aberrant endothelial secretome. Kidney Int..

[73] Gullu H., Erdogan D., Tok D., Topcu S., Caliskan M., Ulus T., Muderrisoglu H. (2005). High serum bilirubin concentrations preserve coronary flow reserve and coronary microvascular functions. Arterioscler. Thromb. Vasc. Biol..

[74] Amor A.J., Ortega E., Perea V., Cofan M., Sala-Vila A., Nunez I., Gilabert R., Ros E. (2017). Relationship Between Total Serum Bilirubin Levels and Carotid and Femoral Atherosclerosis in Familial Dyslipidemia. Arterioscler. Thromb. Vasc. Biol..

[75] Higashi Y., Maruhashi T., Noma K., Kihara Y. (2014). Oxidative stress and endothelial dysfunction: Clinical evidence and therapeutic implications. Trends Cardiovasc. Med..

[76] Maruhashi T., Soga J., Fujimura N., Idei N., Mikami S., Iwamoto Y., Kajikawa M., Matsumoto T., Kihara Y., Chayama K. (2012). Hyperbilirubinemia, augmentation of endothelial function, and decrease in oxidative stress in Gilbert syndrome. Circulation.

[77] Vogel M.E., Idelman G., Konaniah E.S., Zucker S.D. (2017). Bilirubin Prevents Atherosclerotic Lesion Formation in Low-Density Lipoprotein Receptor-Deficient Mice by Inhibiting Endothelial VCAM-1 and ICAM-1 Signaling. J. Am. Heart Assoc..

[78] Gupta N., Singh T., Chaudhary R., Garg S.K., Sandhu G.S., Mittal V., Gupta R., Bodin R., Sule S. (2016). Bilirubin in coronary artery disease: Cytotoxic or protective?. World J. Gastrointest. Pharmacol. Ther..

[79] Radi R. (2018). Oxygen radicals, nitric oxide, and peroxynitrite: Redox pathways in molecular medicine. Proc. Natl. Acad. Sci. USA.

[80] Kaur H., Hughes M.N., Green C.J., Naughton P., Foresti R., Motterlini R. (2003). Interaction of bilirubin and biliverdin with reactive nitrogen species. FEBS Lett..

[81] Johnson J.L. (2014). Emerging regulators of vascular smooth muscle cell function in the development and progression of atherosclerosis. Cardiovasc. Res..

[82] Bennett M.R., Sinha S., Owens G.K. (2016). Vascular Smooth Muscle Cells in Atherosclerosis. Circ. Res..

[83] Ollinger R., Bilban M., Erat A., Froio A., McDaid J., Tyagi S., Csizmadia E., Graca-Souza A.V., Liloia A., Soares M.P. (2005). Bilirubin: A natural inhibitor of vascular smooth muscle cell proliferation. Circulation.

[84] Peyton K.J., Shebib A.R., Azam M.A., Liu X.M., Tulis D.A., Durante W. (2012). Bilirubin inhibits neointima formation and vascular smooth muscle cell proliferation and migration. Front. Pharmacol..

[85] Stoeckius M., Erat A., Fujikawa T., Hiromura M., Koulova A., Otterbein L., Bianchi C., Tobiasch E., Dagon Y., Sellke F.W. (2012). Essential roles of Raf/extracellular signal-regulated kinase/mitogen-activated protein kinase pathway, YY1, and Ca^2+^ influx in growth arrest of human vascular smooth muscle cells by bilirubin. J. Biol. Chem..

[86] Zhang M., Malik A.B., Rehman J. (2014). Endothelial progenitor cells and vascular repair. Curr. Opin. Hematol..

[87] Ozkok A., Yildiz A. (2018). Endothelial Progenitor Cells and Kidney Diseases. Kidney Blood Press. Res..

[88] Jabarpour M., Siavashi V., Asadian S., Babaei H., Jafari S.M., Nassiri S.M. (2018). Hyperbilirubinemia-induced pro-angiogenic activity of infantile endothelial progenitor cells. Microvasc. Res..

[89] Ikeda Y., Hamano H., Satoh A., Horinouchi Y., Izawa-Ishizawa Y., Kihira Y., Ishizawa K., Aihara K., Tsuchiya K., Tamaki T. (2015). Bilirubin exerts pro-angiogenic property through Akt-eNOS-dependent pathway. Hypertens. Res..

[90] Park S., Kim D.H., Hwang J.H., Kim Y.C., Kim J.H., Lim C.S., Kim Y.S., Yang S.H., Lee J.P. (2017). Elevated bilirubin levels are associated with a better renal prognosis and ameliorate kidney fibrosis. PLoS ONE.

[91] Liu J., Dong H., Zhang Y., Cao M., Song L., Pan Q., Bulmer A., Adams D.B., Dong X., Wang H. (2015). Bilirubin Increases Insulin Sensitivity by Regulating Cholesterol Metabolism, Adipokines and PPARgamma Levels. Sci. Rep..

[92] DiNicolantonio J.J., McCarty M.F., O’Keefe J.H. (2018). Antioxidant bilirubin works in multiple ways to reduce risk for obesity and its health complications. Open Heart.

[93] Hull T.D., Agarwal A. (2014). Bilirubin: A potential biomarker and therapeutic target for diabetic nephropathy. Diabetes.

[94] Kim H.J., Vaziri N.D. (2010). Contribution of impaired Nrf2-Keap1 pathway to oxidative stress and inflammation in chronic renal failure. Am. J. Physiol. Renal Physiol..

[95] Qaisiya M., Coda Zabetta C.D., Bellarosa C., Tiribelli C. (2014). Bilirubin mediated oxidative stress involves antioxidant response activation via Nrf2 pathway. Cell. Signal..

[96] Kim S.D., Antenos M., Squires E.J., Kirby G.M. (2013). Cytochrome P450 2A5 and bilirubin: Mechanisms of gene regulation and cytoprotection. Toxicol. Appl. Pharmacol..

[97] Yang T., Richards E.M., Pepine C.J., Raizada M.K. (2018). The gut microbiota and the brain-gut-kidney axis in hypertension and chronic kidney disease. Nat. Rev. Nephrol..

[98] Hamoud A.R., Weaver L., Stec D.E., Hinds T.D. (2018). Bilirubin in the Liver-Gut Signaling Axis. Trends Endocrinol. Metab..

[99] Sakoh T., Nakayama M., Tanaka S., Yoshitomi R., Ura Y., Nishimoto H., Fukui A., Shikuwa Y., Tsuruya K., Kitazono T. (2015). Association of serum total bilirubin with renal outcome in Japanese patients with stages 3-5 chronic kidney disease. Metab. Clin. Exp..

[100] Wu B., Bell K., Stanford A., Kern D.M., Tunceli O., Vupputuri S., Kalsekar I., Willey V. (2016). Understanding CKD among patients with T2DM: Prevalence, temporal trends, and treatment patterns-NHANES 2007-2012. BMJ Open Diabetes Res Care.

[101] Zhu B., Wu X., Bi Y., Yang Y. (2017). Effect of bilirubin concentration on the risk of diabetic complications: A meta-analysis of epidemiologic studies. Sci. Rep..

[102] Kwon Y.J., Lee Y.J., Park B.J., Hong K.W., Jung D.H. (2018). Total serum bilirubin and 8-year incident type 2 diabetes mellitus: The Korean Genome and Epidemiology Study. Diabetes Metab..

[103] Okada H., Fukui M., Tanaka M., Matsumoto S., Kobayashi K., Iwase H., Tomiyasu K., Nakano K., Hasegawa G., Nakamura N. (2014). Low serum bilirubin concentration is a novel risk factor for the development of albuminuria in patients with type 2 diabetes. Metab. Clin. Exp..

[104] Riphagen I.J., Deetman P.E., Bakker S.J., Navis G., Cooper M.E., Lewis J.B., de Zeeuw D., Lambers Heerspink H.J. (2014). Bilirubin and progression of nephropathy in type 2 diabetes: A post hoc analysis of RENAAL with independent replication in IDNT. Diabetes.

[105] Chen Y.H., Hung S.C., Tarng D.C. (2011). Serum bilirubin links UGT1A1*28 polymorphism and predicts long-term cardiovascular events and mortality in chronic hemodialysis patients. Clin. J. Am. Soc. Nephrol..

[106] Fukui M., Tanaka M., Yamazaki M., Hasegawa G., Nishimura M., Iwamoto N., Ono T., Imai S., Nakamura N. (2011). Low serum bilirubin concentration in haemodialysis patients with Type 2 diabetes. Diabet. Med..

[107] Do Sameiro-Faria M., Kohlova M., Ribeiro S., Rocha-Pereira P., Teixeira L., Nascimento H., Reis F., Miranda V., Bronze-da-Rocha E., Quintanilha A. (2014). Potential cardiovascular risk protection of bilirubin in end-stage renal disease patients under hemodialysis. BioMed Res. Int..

[108] Zelenka J., Dvorak A. (2016). Hyperbilirubinemia Protects against Aging-Associated Inflammation and Metabolic Deterioration. Oxid. Med. Cell. Longev..

[109] Tosevska A., Moelzer C., Wallner M., Janosec M., Schwarz U., Kern C., Marculescu R., Doberer D., Weckwerth W., Wagner K.H. (2016). Longer telomeres in chronic, moderate, unconjugated hyperbilirubinaemia: Insights from a human study on Gilbert’s Syndrome. Sci. Rep..

[110] Chmielewski P., Strzelec B., Chmielowiec J., Chmielowiec K., Borysławski K. (2017). Association of serum bilirubin with longevity: Evidence from a retrospective longitudinal study and cross-sectional data. Anthropol. Rev..

[111] Wang J., Wang B., Liang M., Wang G., Li J., Zhang Y., Huo Y., Cui Y., Xu X., Qin X. (2018). Independent and combined effect of bilirubin and smoking on the progression of chronic kidney disease. Clin. Epidemiol..

[112] Su H.H., Kao C.M., Lin Y.C., Lin Y.C., Kao C.C., Chen H.H., Hsu C.C., Chen K.C., Peng C.C., Wu M.S. (2017). Relationship between serum total bilirubin levels and mortality in uremia patients undergoing long-term hemodialysis: A nationwide cohort study. Atherosclerosis.

[113] Hosohata K. (2016). Role of Oxidative Stress in Drug-Induced Kidney Injury. Int. J. Mol. Sci..

[114] Mishra J., Mori K., Ma Q., Kelly C., Barasch J., Devarajan P. (2004). Neutrophil gelatinase-associated lipocalin: A novel early urinary biomarker for cisplatin nephrotoxicity. Am. J. Nephrol..

[115] Han W.K., Bailly V., Abichandani R., Thadhani R., Bonventre J.V. (2002). Kidney Injury Molecule-1 (KIM-1): A novel biomarker for human renal proximal tubule injury. Kidney Int..

[116] Hosohata K., Ando H., Fujiwara Y., Fujimura A. (2011). Vanin-1: A potential biomarker for nephrotoxicant-induced renal injury. Toxicology.

[117] Van Slambrouck C.M., Salem F., Meehan S.M., Chang A. (2013). Bile cast nephropathy is a common pathologic finding for kidney injury associated with severe liver dysfunction. Kidney Int..

[118] Aniort J., Poyet A., Kemeny J.L., Philipponnet C., Heng A.E. (2017). Bile Cast Nephropathy Caused by Obstructive Cholestasis. Am. J. Kidney Dis..

[119] Memon N., Weinberger B.I., Hegyi T., Aleksunes L.M. (2016). Inherited disorders of bilirubin clearance. Pediatr. Res..

[120] Lin J.P., Vitek L., Schwertner H.A. (2010). Serum bilirubin and genes controlling bilirubin concentrations as biomarkers for cardiovascular disease. Clin. Chem..

[121] Chen Y.H., Kuo K.L., Hung S.C., Hsu C.C., Chen Y.H., Tarng D.C. (2014). Length polymorphism in heme oxygenase-1 and risk of CKD among patients with coronary artery disease. J. Am. Soc. Nephrol..

[122] Chen Y.H., Hung S.C., Tarng D.C. (2013). Length polymorphism in heme oxygenase-1 and cardiovascular events and mortality in hemodialysis patients. Clin. J. Am. Soc. Nephrol..

[123] Kimpara T., Takeda A., Watanabe K., Itoyama Y., Ikawa S., Watanabe M., Arai H., Sasaki H., Higuchi S., Okita N. (1997). Microsatellite polymorphism in the human heme oxygenase-1 gene promoter and its application in association studies with Alzheimer and Parkinson disease. Hum. Genet..

[124] Lever J.M., Boddu R., George J.F., Agarwal A. (2016). Heme Oxygenase-1 in Kidney Health and Disease. Antioxid. Redox Signal..

[125] Lee E.Y., Lee Y.H., Kim S.H., Jung K.S., Kwon O., Kim B.S., Nam C.M., Park C.S., Lee B.W., Kang E.S. (2015). Association Between Heme Oxygenase-1 Promoter Polymorphisms and the Development of Albuminuria in Type 2 Diabetes: A Case-Control Study. Medicine (Baltim.).

[126] Ozaki K.S., Marques G.M., Nogueira E., Feitoza R.Q., Cenedeze M.A., Franco M.F., Mazzali M., Soares M.P., Pacheco-Silva A., Camara N.O. (2008). Improved renal function after kidney transplantation is associated with heme oxygenase-1 polymorphism. Clin. Transplant..

[127] Saraf S.L., Zhang X., Shah B., Kanias T., Gudehithlu K.P., Kittles R., Machado R.F., Arruda J.A., Gladwin M.T., Singh A.K. (2015). Genetic variants and cell-free hemoglobin processing in sickle cell nephropathy. Haematologica.

[128] Bosma P., Chowdhury J.R., Jansen P.H. (1995). Genetic inheritance of Gilbert’s syndrome. Lancet.

[129] Lin J.P., O’Donnell C.J., Schwaiger J.P., Cupples L.A., Lingenhel A., Hunt S.C., Yang S., Kronenberg F. (2006). Association between the UGT1A1*28 allele, bilirubin levels, and coronary heart disease in the Framingham Heart Study. Circulation.

[130] Lingenhel A., Kollerits B., Schwaiger J.P., Hunt S.C., Gress R., Hopkins P.N., Schoenborn V., Heid I.M., Kronenberg F. (2008). Serum bilirubin levels, UGT1A1 polymorphisms and risk for coronary artery disease. Exp. Gerontol..

[131] Hsieh C.J., Chen M.J., Liao Y.L., Liao T.N. (2008). Polymorphisms of the uridine-diphosphoglucuronosyltransferase 1A1 gene and coronary artery disease. Cell. Mol. Biol. Lett..

[132] Dekker D., Dorresteijn M.J., Welzen M.E.B., Timman S., Pickkers P., Burger D.M. (2018). Parenteral bilirubin in healthy volunteers: A reintroduction in translational research. Br. J. Clin. Pharmacol..

[133] Abraham N.G., Kappas A. (2008). Pharmacological and clinical aspects of heme oxygenase. Pharmacol. Rev..

[134] Liu J., Wang L., Tian X.Y., Liu L., Wong W.T., Zhang Y., Han Q.B., Ho H.M., Wang N., Wong S.L. (2015). Unconjugated bilirubin mediates heme oxygenase-1-induced vascular benefits in diabetic mice. Diabetes.

[135] Hinds T.D., Stec D.E. (2018). Bilirubin, a Cardiometabolic Signaling Molecule. Hypertension.

[136] Lerner-Marmarosh N., Miralem T., Gibbs P.E., Maines M.D. (2008). Human biliverdin reductase is an ERK activator; hBVR is an ERK nuclear transporter and is required for MAPK signaling. Proc. Natl. Acad. Sci. USA.

[137] Gibbs P.E., Miralem T., Lerner-Marmarosh N., Maines M.D. (2016). Nanoparticle Delivered Human Biliverdin Reductase-Based Peptide Increases Glucose Uptake by Activating IRK/Akt/GSK3 Axis: The Peptide Is Effective in the Cell and Wild-Type and Diabetic Ob/Ob Mice. J. Diabetes Res..

[138] Hu Z., Pei G., Wang P., Yang J., Zhu F., Guo Y., Wang M., Yao Y., Zeng R., Liao W. (2015). Biliverdin Reductase A (BVRA) Mediates Macrophage Expression of Interleukin-10 in Injured Kidney. Int. J. Mol. Sci..

[139] Oda S., Fujiwara R., Kutsuno Y., Fukami T., Itoh T., Yokoi T., Nakajima M. (2015). Targeted screen for human UDP-glucuronosyltransferases inhibitors and the evaluation of potential drug-drug interactions with zafirlukast. Drug Metab. Dispos..

[140] LaFleur J., Bress A.P., Rosenblatt L., Crook J., Sax P.E., Myers J., Ritchings C. (2017). Cardiovascular outcomes among HIV-infected veterans receiving atazanavir. AIDS.

[141] Dekker D., Dorresteijn M.J., Pijnenburg M., Heemskerk S., Rasing-Hoogveld A., Burger D.M., Wagener F.A., Smits P. (2011). The bilirubin-increasing drug atazanavir improves endothelial function in patients with type 2 diabetes mellitus. Arterioscler. Thromb. Vasc. Biol..

[142] Cheng X., Lv X., Qu H., Li D., Hu M., Guo W., Ge G., Dong R. (2017). Comparison of the inhibition potentials of icotinib and erlotinib against human UDP-glucuronosyltransferase 1A1. Acta Pharm. Sin. B.

[143] Ben-Amotz R., Bonagura J., Velayutham M., Hamlin R., Burns P., Adin C. (2014). Intraperitoneal bilirubin administration decreases infarct area in a rat coronary ischemia/reperfusion model. Front. Physiol..

